# Study of the Effect of Graphene Content on the Electrical and Mechanical Properties of Aluminium–Graphene Composites

**DOI:** 10.3390/ma18030590

**Published:** 2025-01-28

**Authors:** Beata Smyrak, Marek Gniełczyk

**Affiliations:** Faculty of Nonferous Metals, AGH University of Krakow, 30-059 Krakow, Poland

**Keywords:** graphene, FLG, MSC composite, aluminium-graphene composite, electrical conductivity

## Abstract

The present paper is dedicated to the search for an alternative material based on an aluminum (Al)—few-layer graphene (FLG) composite for use in electrical applications. Due to its excellent properties, graphene has the potential for use in many applications, especially in electronics, electrical engineering, aerospace, and the automotive industry. One area where the properties of graphene can be exploited is in overhead power transmission, where the main challenge at the moment is to reduce transmission losses. The utilization of conductors that exhibit superior electrical conductivity is instrumental in ensuring the mitigation of transmission losses. The utilization of graphene or other carbon allotropes is appealing due to their elevated electrical conductivity, substantial mechanical strength, and considerable heat resistance, which can enhance the properties of the composite, thereby increasing its resistance to operational conditions, particularly long-term exposure to temperature, a parameter closely related to the current carrying capacity of the OHL. This article presents the findings of research on the production of a composite based on aluminum powder and graphene, as well as the identification of its electrical and mechanical properties. The primary challenge in this research lies in the development of a method to synthesize carbon materials with aluminum using powder metallurgy, with particular attention paid to the mixing and compacting process, which is of significant importance in ensuring the appropriate distribution of carbon material in the composite. The research carried out has determined the influence of the graphene content (0.1–1 wt.%) on the electrical conductivity (max. 35.4 MS/m) and mechanical properties of Al-FLG composites (UTS = 156 MPa).

## 1. Introduction

Allotropic forms of carbon such as graphene possess a range of unique properties that offer significant potential for the creation of MMCs. These are next-generation composite materials with a metallic matrix, which are gaining popularity due to their ability to achieve exceptional strength and operational properties, including rheological and thermal resistance. These composites are formed by the introduction of nonmetallic microparticles and nanoparticles into a metal matrix, resulting in significant changes in both physical and functional properties, allowing for a wide range of applications. In the case of aluminum, MMC-type compounds are particularly popular for improving strength properties using reinforcing particles such as Al_2_O_3_, SiC, AlN, or Y_2_O_3_ [1,2,3].

Graphene and graphene composites, due to their above-standard properties, have already been used in the following applications:

- Thin flexible screens, LED and OLED displays, and electronic paper.
- Processors—new graphene microprocessors will be smaller, more economical, and much faster than silicon-based chips.
- Transistors that operate at frequencies to 1 TH.
- Low-loss power conductors.
- Supercapacitors, which can supply large currents to electrical devices in a short time.

The aforementioned applications present a series of scientific challenges pertaining to the establishment of a permanent bond between graphene and matrix. Primarily, it is imperative to acknowledge the unique structure of the material under consideration, which consists of a single atom of thickness. In the context of aluminum–graphene composites, the primary challenge lies in the development of a synthesis method that facilitates the formation of a permanent bond between graphene and aluminum, whilst preserving the superior properties of the composite.

In all composites, at the interfacial surface, a connection occurs between the matrix and the reinforcing phase, usually in the form of an interfacial compound. In the case of aluminum–graphene composites, a brittle compound, aluminum carbide (Al_4_C_3_), is formed at the interfacial boundaries, often leading to spontaneous cracking of the material. The mechanism of formation of aluminum carbide is not fully understood, and there are conflicting hypotheses in the literature. Some researchers propose that the reaction occurs directly, forming aluminum carbide through nucleation and growth, while others suggest intermediate stages with the formation of thermodynamically unstable compounds leading to Al_4_C_3_ [4,5,6,7,8,9,10,11,12,13,14].

Most methods used to synthesize aluminum–graphene composites involve high-temperature processes, which can directly affect matrix oxidation or reactions at the metal–carbon interface. In their research, Yi Huang and colleagues [8] created aluminum composites with 5 wt.% graphene by weight using a mix sequence, room temperature pressing, and high-pressure torsion (HPT), which was performed at temperatures of 25, 100, and 200 °C for different twist and rotating sequences. At the same time, the composites produced exhibited higher electrical conductivity than aluminum, reaching 66.7% and 64.9% IACS at room temperature. Furthermore, an increase in processing temperature to 200 °C resulted in an increase in electrical conductivity to 69.5% IACS. Bartolucci et al. applied similar mixing techniques, as in [8], in their research [15]. They produced composites with a graphene content of 0.1 wt.%. The materials were obtained by ball milling, hot isostatic pressing (HIP) at 550 °C, and subsequent extrusion. Stearic acid was used to minimize the agglomeration of carbon material during the mixing process. The consolidated powders were subjected to HIP at 550 °C, followed by extrusion. The addition of graphene resulted in a decrease of approximately 18% (262 MPa) compared to pure aluminum (340 MPa) [15]. In their study [16], Shin, HJ Choi, and others aimed to assess the influence of different carbon additives, such as few-layer graphene (FLG), on the strength of the composite. Aluminum powders with a chemical purity of 99.5% FLG were used to create the composites. FLG was obtained by wet mechanical exfoliation followed by mixing with aluminum powder using a planetary mill and stearic acid. The volume fractions were 0.1%, 0.2%, and 0.3% by weight. The synthesis process involved placing the milled powders in a copper tube, densification, and hot rolling at 500 °C. FLG, with its much larger specific surface area, resulted in a tensile strength of approximately 440 MPa with the addition of 0.3% by weight FLG, representing an increase of more than 71% compared to pure aluminum [16].

Generally, on the basis of a literature analysis regarding graphene synthesis methods, it can be observed that graphene as a reinforcing material in high-performance aluminum matrix composites has been used in various forms, including graphene oxide (GO) [13,15], graphite oxide exfoliated into graphene sheets, few-layer graphene (FLG) [16], graphene nanofragments (GNFs) [17,18,19,20], GNP [18,20,21], and graphene. The graphene content in the aluminum matrix composites ranged from 0.1% to 5% by weight. The higher content of the carbon material resulted in a drastic deterioration of the mechanical properties and was not explored extensively. No single method dominated the synthesis of aluminum–graphene composites, as the authors often combined chemical and physical methods, including powder metallurgy and liquid metallurgy. Powder metallurgy, particularly ball milling, was commonly used due to its ease of handling and low processing costs. The process was designed to achieve a uniform distribution of the reinforcement material within the aluminum matrix and increase the specific surface area of the aluminum particles. Various agents, such as polyvinyl alcohol (PVA), stearic acid, and cationic surfactant (CTAB), were utilized to improve wettability and dispersion during the mixing process. Heating processes, typically at 550 °C, were applied to enhance the properties of the composite. Consolidation methods included hot isostatic pressing (HIP), high-pressure torsion (HPT), and hot extrusion. Some studies also employed unconventional approaches, such as the use of copper nanoparticles as a catalytic intermediate or simultaneous frictional milling and dispersion of graphene during mechanical mixing. The addition of graphene generally led to an increase in tensile strength, ranging from 11% to 206% compared to pure aluminum, depending on the synthesis method and the graphene content. However, there were exceptions, such as the study by Bartolucci et al. [15], where even a weight-increasing 0.1% of carbon material resulted in a 23% decrease in composite strength compared to pure aluminum processed under similar conditions. This decrease was attributed to the formation of brittle aluminum carbide during composite processing at high temperatures or to the poor quality of graphene. In the literature, a trend was observed in which an increase in graphene content initially enhanced mechanical properties, reaching a maximum boundary value depending on the processing method, followed by a sharp decrease in tensile strength along with a reduction in accompanied deformability [14,22]. This observed trend was often attributed to excessive carbon material content, leading to uneven distribution within the matrix and the formation of carbon agglomerates. However, there were cases where the addition of graphene in the form of graphene nanofragments (GNFs) at 0.5% by weight allowed the acquisition of a compound characterized by a higher tensile strength (approximately 18%), while maintaining higher elongation values (approximately 15%) compared to pure aluminum. However, increasing the graphene content to 1% by weight still resulted in improved tensile strength (69%) but a 52% reduction in the deformability of the composite compared to pure aluminum [17]. Methods based on powder metallurgy typically involve carbon materials in the form of graphene and carbon nanotubes, and graphene flake material is commonly used [17,18,19,23,24,25,26,27].

Based on the synthesis of the literature analysis of aluminum graphene, all data on synthesis methods and their effects in terms of mechanical properties are presented in Table 1. Specifically, the tables include the results of tensile strength, elongation, and percentage changes in composite strength compared to aluminum.

It can be observed that the carbon material content in the composites generally ranged up to 5 wt.%. At the same time, it is noticeable that composites with graphene content not exceeding 1% by weight are the most popular among researchers [10,11,12,13,14,15,16,22]. The highest tensile strength value (440 MPa) and elongation (5.5 wt.%) were achieved in the case of the composites produced by Shin and colleagues [16]. These composites were manufactured using powder metallurgy technology, high-energy ball milling, and then densification by hot rolling at a temperature of 500 °C. The FLG content in the composite with the highest tensile strength was 0.3–0.5 wt.%. The addition of carbon material allowed the researchers to obtain a composite whose tensile strength, relative to the strength of the aluminum matrix alone, increased by almost 70%, with a recorded decrease in elongation from 13% to 5.5%, representing a reduction of about 60% [19,23,24,25,26,27].

In addition to powder metallurgy, other fabrication methods that enable the production of aluminum–graphene composites include liquid metallurgy (casting processes), plastic processing methods (rolling, wire drawing, serve plastic deformation (SPD)), additive manufacturing, friction stir processing (FSP), and electrodeposition methods [20,21,28,29,30]. For instance, the outcomes of a study [30] demonstrated that Al-C composites fabricated by the FSP process achieved an electrical conductivity of 34.12 MS/m and an ultimate tensile strength (UTS) of 14.9 MPa. Furthermore, in this case, no cracks, voids, or aluminum carbide (Al_3_C_4_) were found between the graphene and aluminum, indicating that clean and intimate contact interfaces were formed under thermomechanical conditions during the fabrication process. On the other hand, there has been an increasing tendency to utilize recycled materials as raw materials in research. In a study [30], aluminum (recycled)–graphene composites with 0.5 wt.% graphene were obtained with an alloy electrical conductivity of 36.8 MS/m, and their tensile strength was determined to be 180 MPa after 90% cold rolling and aging at 200 Ԩ for 1 h [30].

In summary, powder metallurgy is one of the most common methods of weathering aluminum–graphene composites nowadays.

## 2. Research Objectives

The exceptional properties of graphene, in particular its electrical, thermal, and mechanical characteristics, make it an intriguing material component, especially for electrical applications such as the construction of power transmission lines. In this case, the challenge lies in developing a method to synthesize carbon materials with aluminum, which is a fundamental material used in power transmission lines.

A review of the literature indicates that the most popular method among scientists for synthesizing such materials is the mechanical synthesis of a powder mixture, which includes processes such as (a) mixing, (b) compacting, (c) sintering, and (d) consolidation. The crucial stage in this case is the mixing and compacting process, which should ensure the homogeneity of the mixture, i.e., the appropriate distribution of carbon material in the composite, along with compacting and further processing of the final product.

The aim of this study is to determine the effect of graphene content on the electrical conductivity and mechanical properties of aluminum and graphene composites obtained by mechanical synthesis.

## 3. Materials, Methods, and Equipment

On the basis of the literature analysis, mechanical synthesis was chosen for this experimental research on the synthesis of aluminum–graphene composites. The selection of this method was driven by the goal of the study, which was to produce an Al-C composite in the form of a rod that serves as a fundamental component for overhead line conductors.

### 3.1. Materials

**Figure 1:**
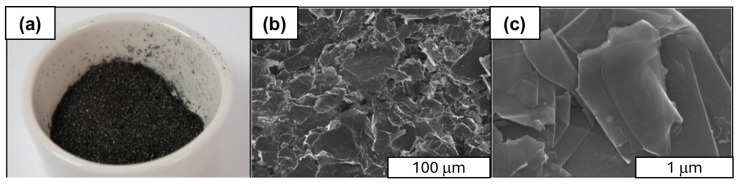
Compilation of scanning electron microscope images of graphene used in the synthesis: (**a**) surface, (**b**) at a magnification of ×500, and (**c**) at a magnification of ×50,000.

Flake graphene (FLG) and spherical aluminum powder were utilized in the synthesis process of the composites. The form of graphene used for the synthesis of aluminum graphene is presented in Figure 1.

The graphene used for the synthesis consisted of flake graphene (FLG) with properties presented in Table 1.

**Figure 2:**
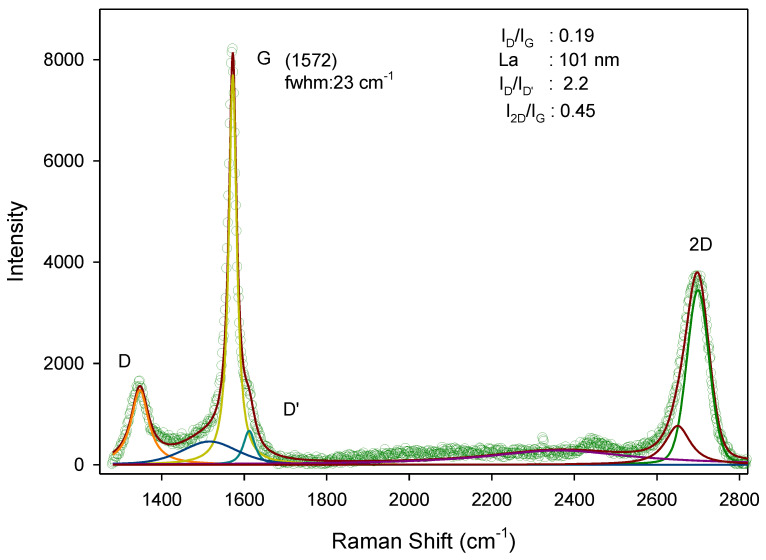
Raman spectrum that reveals the characteristics of the carbon material.

The graphene was also characterized using Raman spectroscopy, which involves measuring Raman scattering radiation. The results of the Raman spectrum are presented in Figure 2.

**Figure 3:**
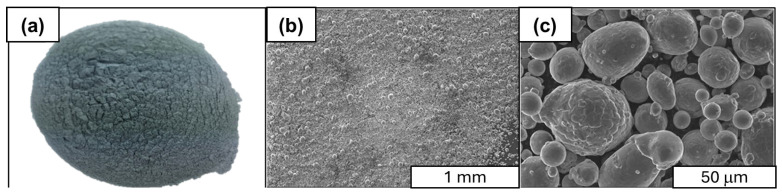
The SEM images of aluminum powder used in the synthesis: (**a**) surface of aluminum powder; (**b**,**c**) SEM images of aluminum powder surface; (**b**)—magnification: 50×; (**c**)—magnification: 1000×.

This research used spherical powders, which were formed by introducing irregularly shaped powders into a plasma area, where they melted in a protective atmosphere and then solidified, taking on a spherical shape. The technical purity of the powders was 99.8%, with a medium grain size of about 5 micrometers. Figure 3 presents a graphical compilation of images of spherical aluminum powder visible to the naked eye and by scanning microscopy.

**Figure 4:**
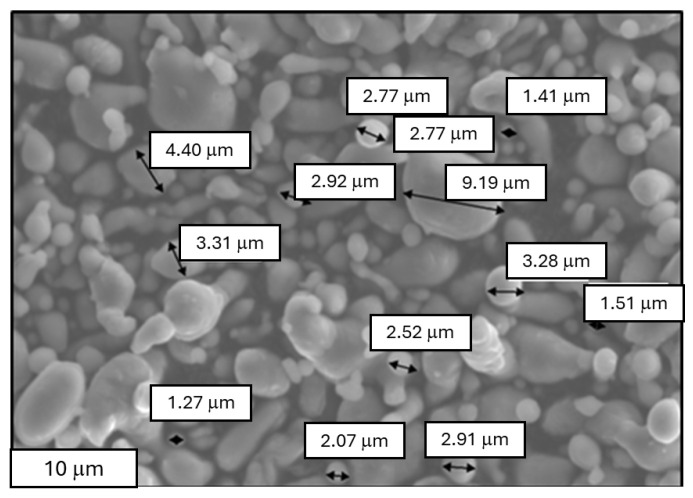
The SEM image of aluminum powder, with grain size measurements, magnification 2000×.

For a detailed analysis, the spherical aluminum powder was examined using scanning microscopy (see Figure 4). The investigations were conducted on loosely scattered powders, as well as on compacted powders, to reveal their chemical composition, morphology, and particle size.

### 3.2. Methods of Aluminum–Graphene Composite Manufacturing

**Figure 5:**
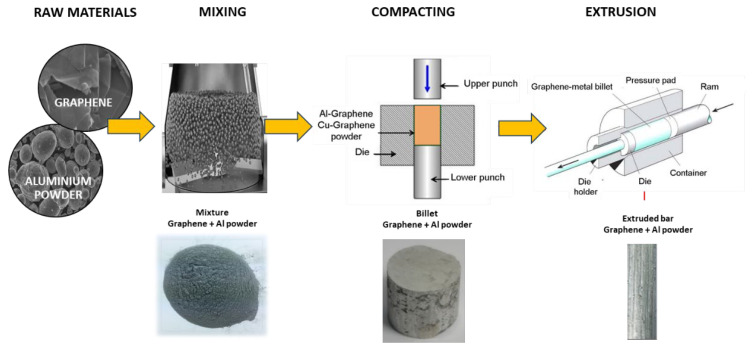
Schematic representation of the manufacturing of aluminum–graphene composites.

The method of producing aluminum–graphene composites was based on mechanical synthesis, which consisted of the following processes: (a) powder mixing process, (b) compacting, and (c) consolidation in the process of extruding rods (see Figure 5).

**Figure 6:**
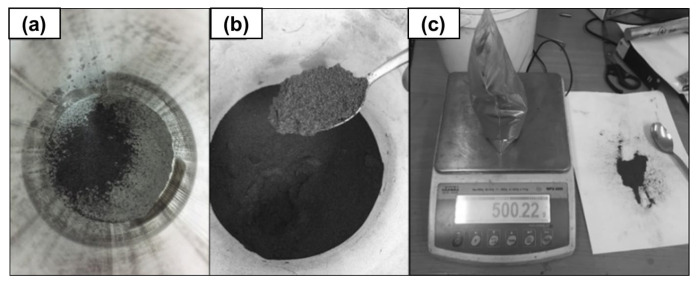
Preparing the appropriate FLG content and introducing it into aluminum powder, (**a**) FLG, (**b**) aluminum powder, (**c**) mixing of FLG and aluminum powder.

Mixtures with varying percentages of carbon material in the form of graphene were prepared. Powder weights with the correct percentage compositions were prepared using an analytical balance, as illustrated in Figure 6.

**Figure 7:**
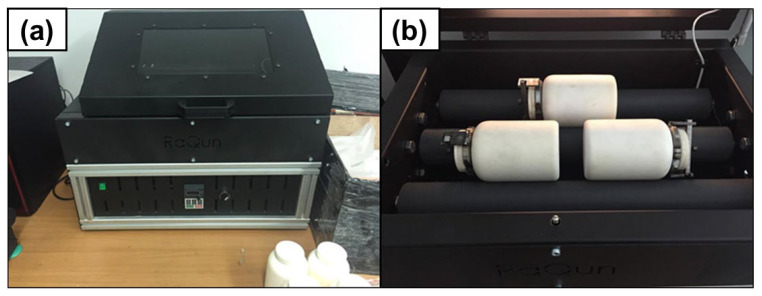
Turbula mixer for mixing process, (**a**)—general view of the device, (**b**)—internal view of the device.

As a result of preliminary research, several variants of the mixing process were developed using ball mills and turbines, allowing a homogeneous distribution of carbon materials throughout the entire volume of the matrix, consisting of spherical aluminum powder. The devices used for the mixing process are presented in Figure 7.

The mixing process of a mixture of aluminum powder with FLG, in proportions of 0, 0.1, 0.2, 0.5, and 1 wt.%, was conducted in a turbula mixer for a duration of 30–60 min with a rotation of 80 rpm. The prepared powder mixtures were weighed using a laboratory analytical balance, and the mixing aluminum–graphene samples obtained after 60 min of mixing were selected for further studies. The detailed parameters under which the composite powder blends were prepared are presented in Table 2. In the subsequent step, the mixture of aluminum powder and graphene underwent a pressing process, which occurred with a hydraulic press, with the compaction of the powder inside the container being achieved through the vertical movement of the press piston. The composition of each individual charge within the press chamber consisted of a precise mixture of aluminum powder and carbon material, with a total weight of approximately 100 g. The pressing operation was carried out by applying a pressure of 30 atm to the compressed powder mixture (refer to Table 2 for further details).

The pressing process was employed to yield four aluminum–graphene composites with varying graphene content, with a reference sample composed of aluminum also prepared for comparative analysis. Subsequent to the pressing process, the composites adopted a compact form with a diameter of 40 mm and a height of approximately 40 mm. 

**Figure 8:**
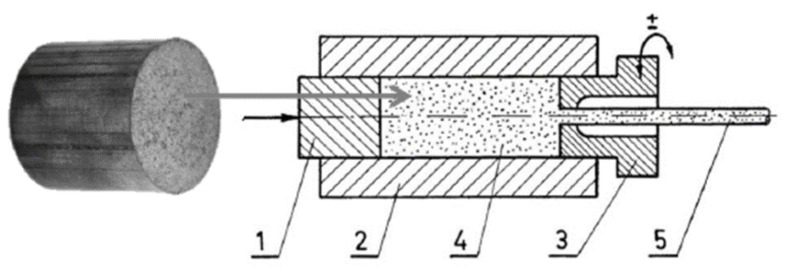
Extrusion with rotating die: (1) press ram, (2) recipient sleeve, (3) cyclically rotating die, (4) feedstock material, and (5) finished product.

In the subsequent phase of the investigation, the compacts were subjected to unconventional extrusion using a rotating die. This method entails the utilization of a movable die that oscillates cyclically around its own axis at a predetermined angle and frequency. The movement of the die engenders a modification in the trajectory of the plastic deformation of the extruded material, thereby enabling substantial deformation. A schematic of the extrusion process is depicted in Figure 8.

Extrusion tests were conducted with the recipient sleeve and feedstock material heated to a temperature of 290 °C. Each time, the feedstock material was preheated in the press recipient for approximately 10 min before the extrusion process started. Detailed parameters of extrusion were presented in Table 3.

### 3.3. Equipment

A Jeol JXA 8230 microanalyzer (JEOL Ltd., Tokyo, Japan) was utilized for the analysis of powders and mixtures, with an acceleration voltage of 15 kV and an electron beam current of 30 nA employed during the testing process. The measurements were conducted employing energy-dispersive spectrometry (EDS) and wavelength-dispersive spectrometry (WDS). The wavelength-dispersive method has been shown to facilitate more precise measurement of light elements, a property that is also characterized by enhanced element detection and a higher resolution of spectral lines. This study encompassed both powder and solid sample testing methodologies.

The study of the fracture of the Al-FLG composite rods after tensile testing was carried out using a Hitachi SU-70 high-resolution scanning electron microscope (SEM), Hitachi, Tokyo, Japan, which has two types of detectors, i.e., a secondary electron detector (SE) and a backscattered electron detector (BSE), and a characteristic radiation detector EDX (energy-dispersive X-ray spectroscopy). The accelerating voltages (AVs) were set at 15 kV.

Raman spectroscopy was used to evaluate the quality of the graphene-based material. The study was performed using a Renishaw inVia confocal Raman microscope (Renishaw, Gloucestershire, UK) with the following specifications: excitation (cw): 532 nm, 633 nm, 1064 nm, and 325 nm laser; configuration: backscattering; spatial resolution: 0.5 µm; resolution: approximately 1.5 cm^−1^; spectral range (Raman shift): 50 cm^−1^, 6000 cm^−1^; experimental temperature: room temperature.

The resistance measurements of Al-C composite rods were performed using a Thomson RESISTOMAT^®^ 2304 bridge from Burster (Gernsbach, Germany), with a maximum measurement resolution of 1 nΩ at measurement currents between 100 µA and 10 A. The accuracy class is 0.01%, according to the manufacturer.

The mechanical properties of the Al-FLG composite rods were determined through a static uniaxial tensile test at an ambient temperature of approximately 20 °C. The gauge length was set at 200 mm, and the tensile speed was adjusted to 50 mm/min. The uniaxial tensile tests were conducted using Zwick Z020 and Z100 testing machines (Zwick, Ulm, Germany).

## 4. Research Results and Discussion

In the preliminary phase, an investigation was conducted to determine the efficacy of the flake graphene and aluminum powder amalgamation process. To this end, two distinct mixtures of spherical aluminum powder and FLG, with concentrations of 0.2 wt.% and 1 wt.%, were selected for analysis. The homogeneity of these mixtures was evaluated by assessing the distribution of carbon material within the aluminum matrix. The analysis encompassed the following:

(a) Energy-dispersive spectroscopy (EDS) mapping of the carbon distribution;
(b) Quantitative analysis of the chemical composition using wavelength-dispersive spectroscopy (WDS);
(c) Mapping distribution of individual elements The ensuing research results are presented for two variants of mixtures: Al–Graphene (0.2 wt.%), and Al–Graphene (1.0 wt.%).

**Figure 9:**
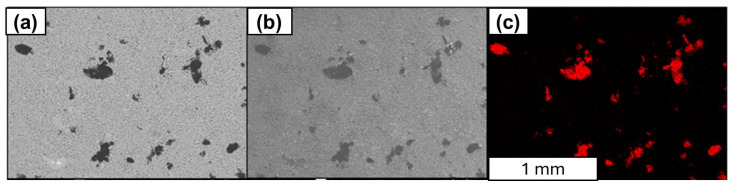
SEM analysis of the distribution of carbon material in Al-FLG (0.2 wt.%), powder mixture (**a**,**b**) and EDS analysis (**c**), magnification: ×50.

The carbon distribution map for the Al-FLG (0.2 wt.%) powder mixture at 50× magnification is shown in Figure 9a,b, and the contrast and unambiguous content of the carbon material can be observed in Figure 9c.

**Figure 10:**
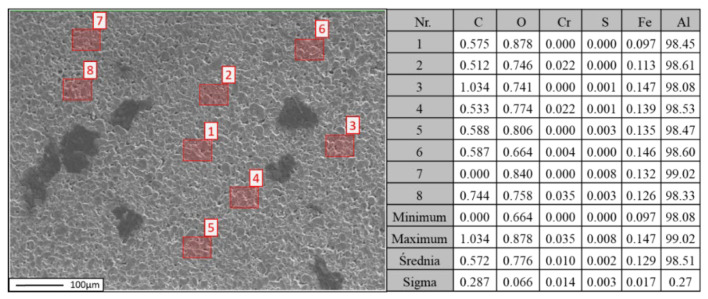
Localization of graphene for WDS analysis of the Al-FLG (0.2 wt.%) powder mixture (**left**) and the results of the chemical composition analysis of the powder mixture Al-FLG (0.2 wt.%) (**right**)—measurement no 1.

**Figure 11:**
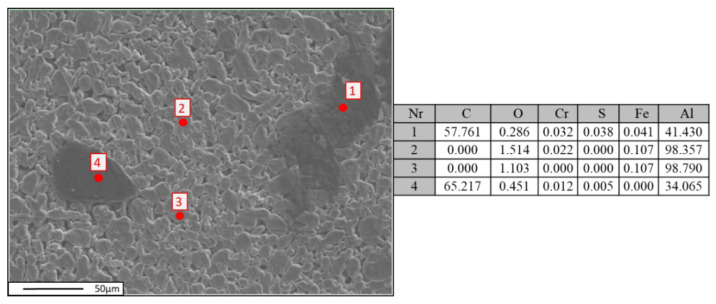
Localization of graphene for WDS analysis of the Al-FLG (0.2 wt.%) powder mixture (**left**) and results of the chemical composition analysis of the powder mixture Al–graphene (0.2 wt.%) (**right**)—measurement no 2.

**Figure 12:**
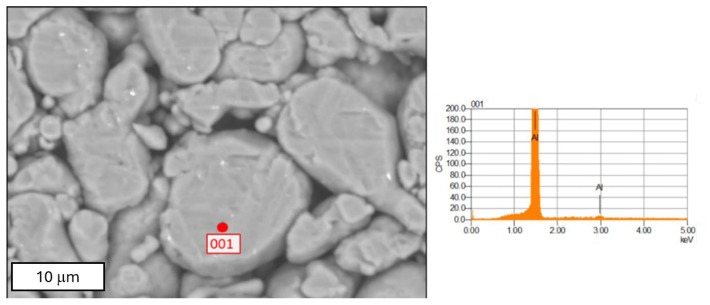
Localization of points for chemical composition measurement using the EDS method (**on the left**); images of the characteristic spectrum of powder mixture Al-FLG (0.2 wt.%), magnification 2000×.

Quantitative analysis of the chemical composition was performed using wavelength-dispersive spectroscopy (WDS). In Figure 10, Figure 11 and Figure 12, the locations of the points selected for the chemical composition analysis of the Al-FLG mixture (0.2 wt.%) are presented, along with the results of the analysis. Furthermore, Figure 10 shows the obtained EDS analysis spectrum obtained for the examined area.

**Figure 13:**
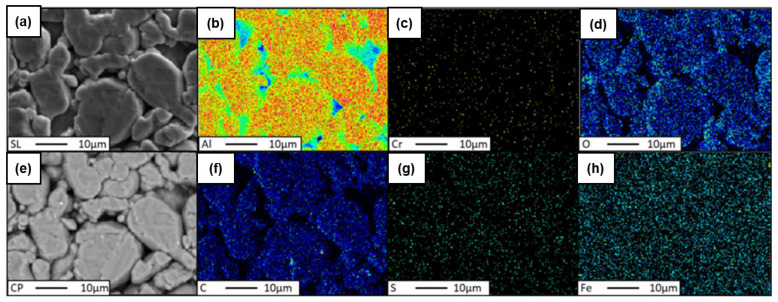
Elemental distribution maps for region No. 2, Al-FLG (0.2 wt.%), magnification 2000×; (**a**) sulfur, (**b**) aluminum, (**c**) chromium, (**d**) oxygen, (**e**) copper, (**f**) carbon, (**g**) sulfur, (**h**) iron.

Based on the analysis of the element distribution map for area 2, the powder mixture of Al-FLG (0.2 wt.%), the measurement taken at point 001 according to the CPS chart indicates that the carbon material was not distributed in the examined area. This is further confirmed in Figure 13.

**Figure 14:**
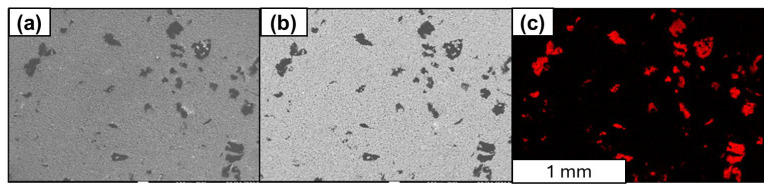
SEM analysis of the distribution of carbon material in Al-FLG (1.0 wt.%), powder mixture (**a**,**b**) and EDS analysis (**c**), magnification: ×50.

The carbon distribution map of the Al-FLG (1.0 wt.%) powder mixture at 50× magnification using EDS is shown in Figure 14. The noticeable increase in the content of FLG from 0.2 to 1 wt.% in the aluminum matrix is evident. The FLG exhibits a nonuniform distribution within the aluminum matrix, suggesting a substantial degree of randomness in its arrangement.

**Figure 15:**
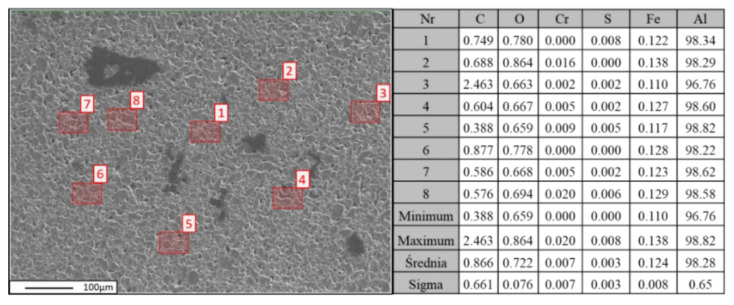
Localization of carbon material and WDS chemical composition analysis of the Al-FLG (1.0 wt.%) powder mixture (**on the left**) and the results of the chemical composition analysis of the Al-FLG (1.0 wt.%) powder mixture for measurement No. 2 (**on the right**).

The results of studying the chemical composition and locating and quantifying the carbon in the matrix are shown in Figure 15.

**Figure 16:**
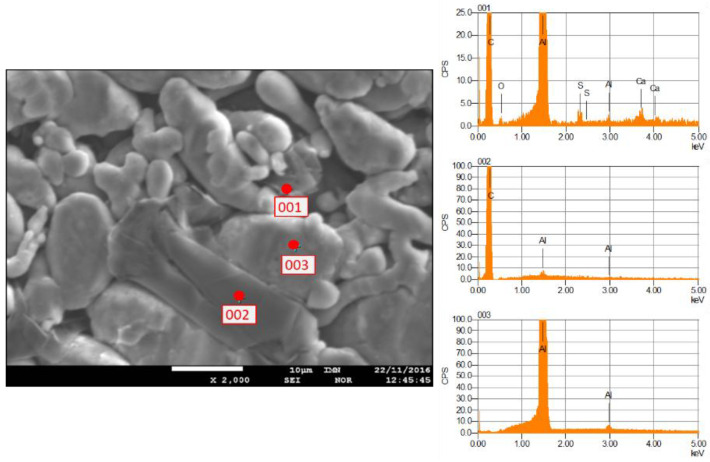
Localization of points for chemical composition measurements using the EDS method magnification ×2000. (**left**) and images of the characteristic spectrum, area no. 1, 2 and 3 of the Al-FLG (1.0 wt.%) mixture (**right**).

**Figure 17:**
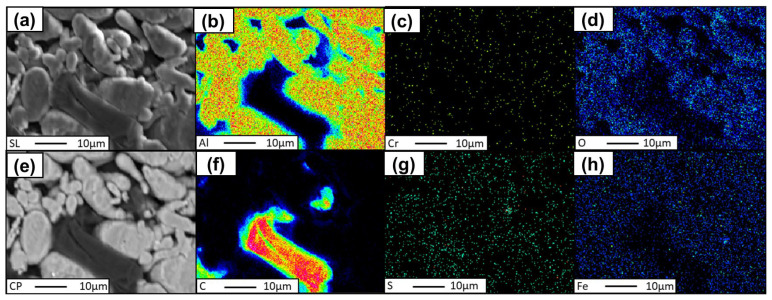
Element distribution map for region no. 1, powder mixture no. 2 Al-FLG (1.0 wt.%), surface 2000×: (**a**) SL, (**b**) Al., (**c**) Cr, (**d**) O, (**e**) CP, (**f**) C, (**g**) S, and (**h**) Fe.

Similarly to the analysis of the Al-FLG (0.2 wt.%) mixture, EDS analyses were performed for the Al-FLG (1.0 wt.%) powder mixture. The results of these analyses produced element distribution maps for two areas of the powder mixture. The measurement results for areas 1 and 2 are presented in Figure 16 and Figure 17.

The EDS analysis revealed the presence of carbon at points designated as 001 and 002 within the examined area, manifested as a folded graphene sheet with dimensions of approximately 10 × 20 μm. In addition to the aforementioned examples, the images of the aluminum and graphene powder mixture generally indicated an even distribution of graphene throughout the material’s volume.

**Figure 18:**
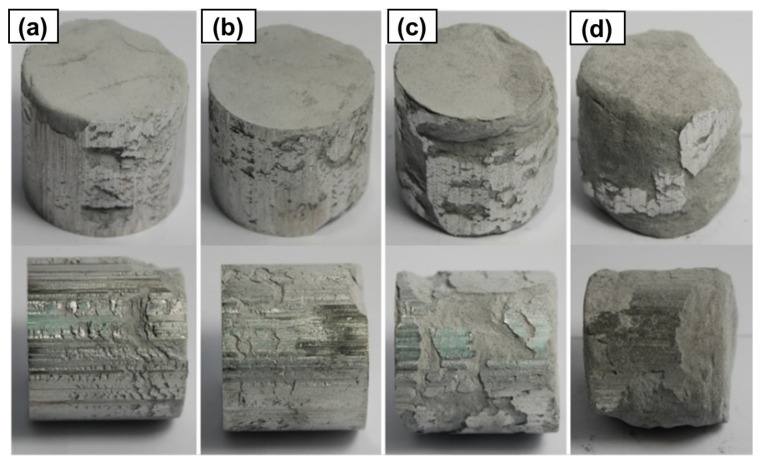
The compacted samples of Al-FLG composites with graphene content of (**a**) 0.1%, (**b**) 0.2%, (**c**) 0.5%, and (**d**) 1 wt.%.

Subsequently to the pressing process, four aluminum–graphene composites with varying graphene content were obtained. For comparison purposes, a reference sample of pure aluminum was also produced. The compacts produced after the pressing process had a diameter of approximately 40 mm and a height of approximately 40 mm. The quality of their surfaces varied, as illustrated in the images included in Figure 18. The quality analysis of these compacts revealed that the compact with the highest graphene content in the aluminum matrix exhibited the greatest brittleness.

**Figure 19:**
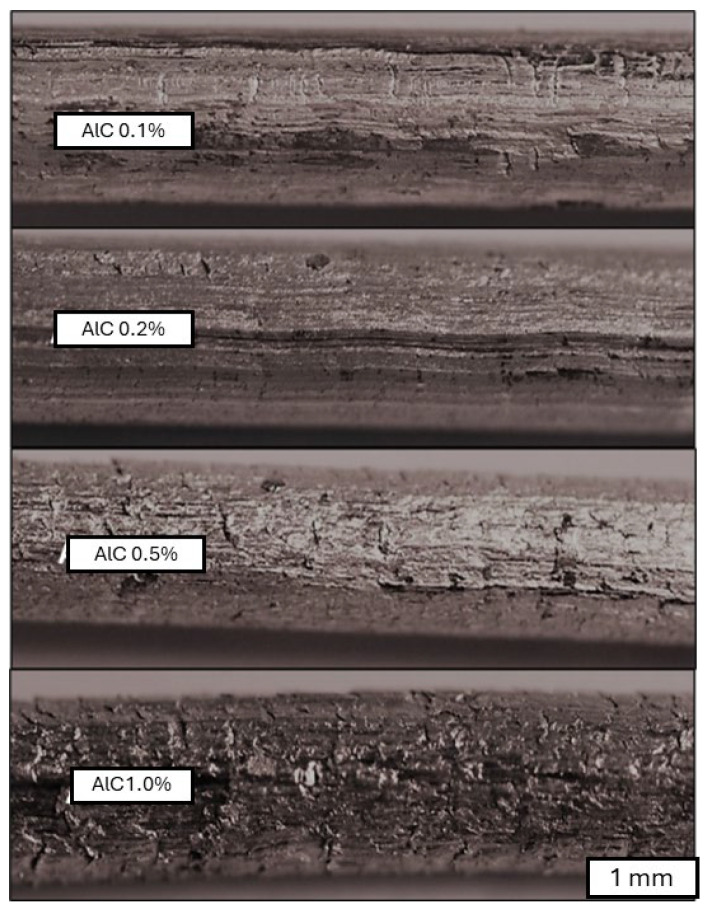
Surface of the aluminum–graphene rods obtained in the extrusion process.

Within the extrusion process using the rotating die, five materials were obtained which were characterized by different carbon material content. The surface quality of the resulting compounds in the form of 4 mm diameter rods is presented in Figure 19. Through visual analysis of the surface quality of the extruded rods, it is evident that with an increase in the amount of carbon material, the final composite exhibits a nonuniform, scale-like surface, which is significant for subsequent plastic processing operations.

Subsequently, the composites thus produced were subjected to further tests to determine their density, electrical conductivity, and mechanical properties. The results of the investigation are presented in Table 4.

In the case of the density measurements of the composites, it was observed that the addition of carbon material to the aluminum matrix did not significantly affect the density difference in the composite rods, which had a density of around 2.65 g/cm^3^. Conversely, the analysis of electrical conductivity of the Al-FLG composites showed that the 0.2 wt.% composite exhibited the highest conductivity, which is approximately 1% lower than the electrical conductivity of a rod made of pure aluminium. The 1.0 wt.% composite Al-FLG exhibited the lowest conductivity of 31.25 MS/m, representing an approximately 20% lower value than the reference sample (pure aluminium). With respect to the mechanical properties, it was observed that the tensile strength generally decreased with an increase in the carbon material content in the aluminium matrix. The highest tensile strength (UTS) of 156 MPa was obtained in the 1.0 wt.% Al-FLG composite, indicating a 5% increase in UTS compared to the reference material. In both cases, the addition of graphene up to 0.5 wt.% did not result in a significant deterioration of the elongation of the composite. However, the addition of graphene with a content of 1 wt.% led to a reduction in the elongation of the composite to 3%, which is approximately 60% lower than that of the reference material.

**Figure 20:**
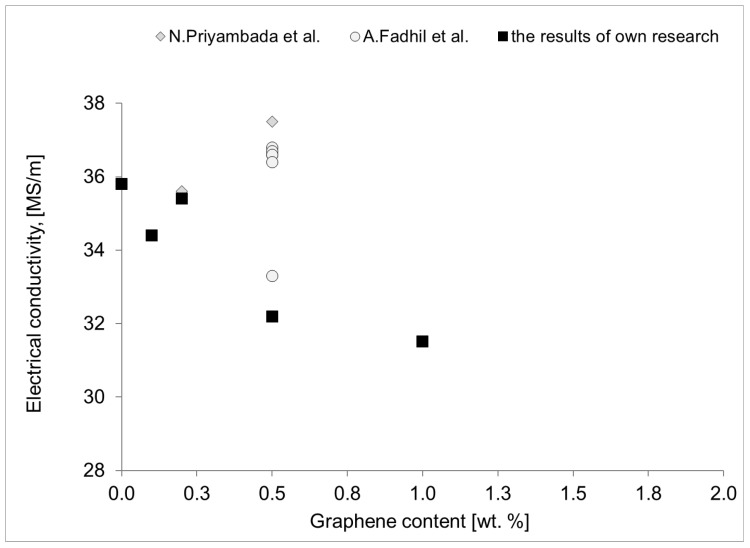
The results of the electrical conductivity of the Al-graphene composites obtained in this work against the results presented in the literature [20,30].

**Figure 21:**
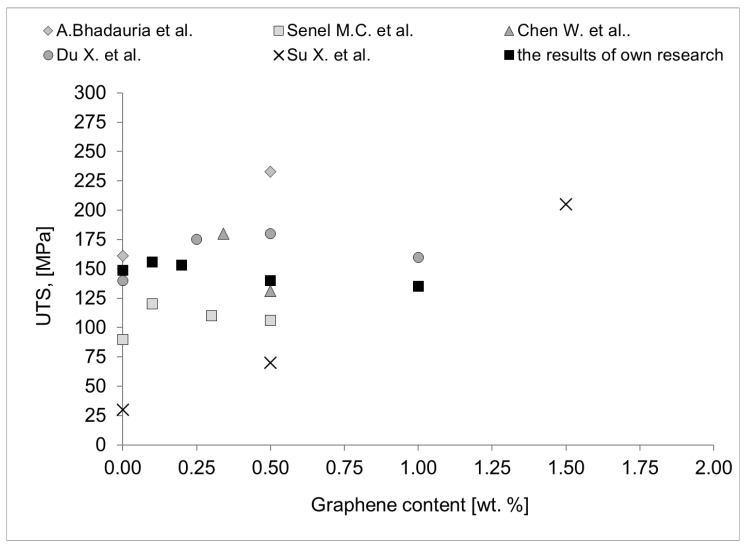
UTS results of Al-graphene composites obtained in this work against the results presented in the literature [10,13,17,20,21,22,31,32,33,34].

As demonstrated in Figure 20 and Figure 21, the quantity of graphene employed is directly proportional to its impact on electrical conductivity (Figure 20) and mechanical strength (Figure 21). The examination of these data indicates that the range of values obtained in the present study is consistent with the range of values reported in the extant literature. From the perspective of the objectives delineated in this study, the developed materials are anticipated to exhibit above-standard properties, such as high tensile strength and high electrical conductivity, concurrently. A thorough analysis of the available data set reveals that the Al-FLG composite rods with a 1 wt.% graphene addition do not exhibit above-standard electrical and strength properties. The obtained values of UTS (156 MPa) and electrical conductivity (35.4 MS/m) are comparable to those of pure aluminum. In the case of the analysis of the electrical properties, no higher values were obtained in relation to aluminum, although several references in the literature show such values. Thus, we conclude that the problem is still poorly understood and requires more fundamental research.

**Figure 22:**
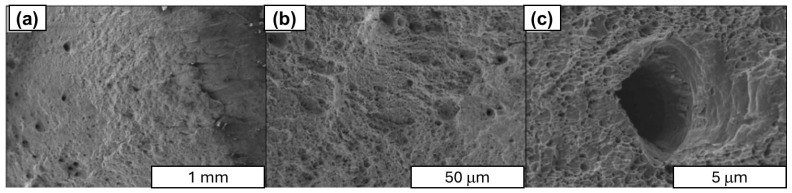
SEM images of the pure aluminum rod fracture surface after uniaxial tensile testing; magnification: (**a**) ×100 SE, (**b**) ×200 SE, (**c**) ×1000 SE.

**Figure 23:**
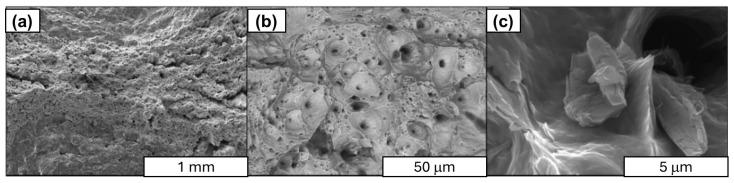
SEM images of the Al-FLG (0.2 wt.%) composite rod fracture surface graphene content; 0.2% magnification; (**a**) ×50, SE, (**b**) ×1000 PDBSE, (**c**) ×10,000 SE.

**Figure 24:**
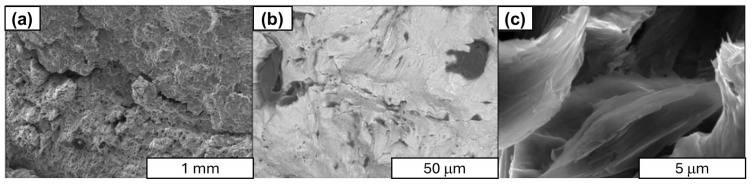
SEM images of the Al-FLG (0.5 wt.%) composite rod fracture surface; magnification: (**a**) ×50, SE, (**b**) ×1000 PDBSE, (**c**) ×10,000 SE.

**Figure 25:**
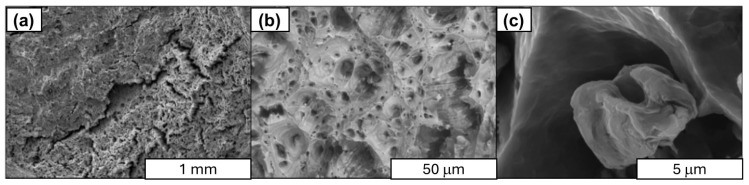
SEM images of the surface of the Al-FLG (1 wt.%); magnification: (**a**) ×50, SE, (**b**) ×1000 PDBSE, (**c**) ×10,000 SE.

In the next stage of research structural studies, the SEM of fracture surfaces obtained during uniaxial tensile testing of aluminum–graphene rods with varying carbon material content (see Figure 22, Figure 23, Figure 24 and Figure 25). Observations were made, which led to the identification of the presence of graphene in the composite. This was found to be in the form of agglomerates of loosely arranged carbon layers that were unconnected to the aluminum matrix. The images clearly demonstrate that there is no durable connection between graphene and the aluminum matrix, as evidenced by numerous fractures in the cross section of the composites, the number of which increases with increasing carbon material content in the composite. For example, in the 1 wt.% Al-FLG composites (see Figure 22), a linear delamination of the composite material was observed, which was revealed during the stretching process. In comparison, the rod displays a conventional plastic fracture, accompanied by the development of flow surfaces.

## 5. Conclusions

The development of a material that combines two opposing properties, for example, high tensile strength and high electrical conductivity, is a rapidly growing research trend in the field of new materials for electrical applications.

1. The Al-FLG composite showed a wide range of electrical properties, depending on the carbon material content (35.4–31.2 MS/m). The most favorable electrical properties (35.4 MS/m) were obtained for composites containing approximately 0.2 wt.% graphene.
2. The Al-FLG composites showed a wide range of mechanical properties depending on the carbon material content. The highest tensile strength (156 MPa) was obtained in composites containing approximately 0.2 wt.% graphene.
3. Al-FLG composites showed a homogeneous distribution of graphene throughout the composite up to 0.2 wt.% graphene content. Increasing the graphene content to 0.2 wt.% resulted in increased inhomogeneity. It was found that the higher content of graphene additives tended to form conglomerates (local clusters) in the aluminum matrix. This phenomenon increased with increasing graphene content in the mixture, leading to increased brittleness (Figure 22, Figure 23, Figure 24 and Figure 25).
4. According to the SEM analysis of the fracture of Al-FLG rods with a graphene content of 1 wt.%, a linear delamination of the composite material was observed, which was revealed during the tensile process (Figure 22, Figure 23, Figure 24 and Figure 25). Compared to the bar, it shows a conventional plastic fracture accompanied by the development of a flow surface.
5. In the case of pronounced susceptibility to graphene agglomeration, which was found to be particularly pronounced at higher graphene contents (above 5%), a solution may be to use a different form of graphene, known as functionalized graphene. The ability to inhibit graphene agglomeration can be achieved by physically or chemically modifying graphene, i.e., modifying the graphene surface with metal nanoparticles (e.g., aluminum). At this stage of the research, important limitations in the synthesis of aluminum and graphene were identified, namely the susceptibility of graphene to agglomeration and the lack of high electrical conductivity of the Al–graphene composite, which was also one of the objectives of this work. In the case of graphene’s pronounced susceptibility to agglomeration, which was found to be particularly pronounced at higher graphene contents (above 5%), another form of graphene, known as functionalized graphene, may be a solution. The ability to inhibit graphene agglomeration can be achieved through the physical or chemical modification of graphene, i.e., the modification of the graphene surface with metal nanoparticles (e.g., aluminum) or the electrodeposition of graphene on aluminum.
6. An ongoing scientific challenge is the development of aluminum–graphene composites that facilitate enhanced electrical conductivity. Key issues in this regard include ensuring contact between the aluminum matrix and the graphene, limiting the formation of different compounds (e.g., Al_2_O_3_ or intermetallic compounds such as Al_4_C_3_) during synthesis, ensuring the structural integrity of the graphene, and ensuring adequate dispersion of the graphene in the aluminum matrix. The results of the literature review suggest that an alternative method that may yield beneficial results is a casting process carried out under the influence of an electric current flow, which will enable the formation of covalent bonds between aluminum and graphene.

## Figures and Tables

**Table 1:** Properties of graphene (FLG) used in this research.

Resistance	Electron Mobility	Density	Electrical Conductivity
R = 0101 Ω/m	μ = 1.05 × 10^3^ cm^2^/Vs	*n* = 4.279 × 10^16^/cm^2^	σ = 41 × 10^3^ S/m

**Table 2:** Synthesis parameters of Al-FLG composites with different graphene content.

Type of Composite	Mixing							Compaction
	Sample No	Powder Mass	Graphene Mass	Graphene Content		Time	Temperature	Pressure
		g	g	wt.%	vol.%	[s]	[°C]	atm
Al-FLG (0.1 wt.%)	1	100	0.1	0.1	0.1	3600	20	30
Al-FLG (0.2 wt.%)	2	100	0.2	0.2	0.2	3600	20	30
Al-FLG (0.5 wt.%)	3	100	0.5	0.5	0.6	3600	20	30
Al-FLG (1.0 wt.%)	4	100	1	1	1.2	3600	20	30

**Table 3:** Extrusion parameters for the extrusion process.

Parameters	Value	Unit
Elongation coefficient	100	[-]
Extrusion force	1000	[kN]
Extrusion speed	0.3	[mm/s]
Angle of die reverse twisting	±8	[°]
Die rotation frequency	5	[Hz]
Initial temperature of the aluminum	290	[°C]
The diameter of the bar obtained	4	[mm]

**Table 4:** Results of testing of the density, electrical conductivity, and mechanical properties of the Al-FLG rods after the extrusion process.

	Density	Resistance	Electrical Conductivity	Proof Stress	UTS	Elongation
	[g/cm^3^]	[mΩ]	[MS/m]	[MPa]	[MPa]	[%]
Al.	2.68	2.27	35.8 ± 0.5	129 ± 4	149 ± 3.5	8.2 ± 0.4
Al-FLG (0.1 wt.%)	2.65	2.36	34.4 ± 0.8	145 ± 3	156 ± 2.8	9.6 ± 0.8
Al-FLG (0.2 wt.%)	2.63	2.29	35.4 ± 0.8	143 ± 6	153 ± 4.2	10.1 ± 0.8
Al-FLG (0.5 wt.%)	2.64	2.53	32.2 ± 1.5	108 ± 8	140 ± 3.0	7.9 ± 1.3
Al-FLG (1.0 wt.%)	2.64	2.47	31.52 ± 2.5	126 ± 8	135 ± 2.6	3 ± 1.5

## Data Availability

The original contributions presented in this study are included in the article. Further inquiries can be directed to the corresponding author.
